# Patterning principles of morphogen gradients

**DOI:** 10.1098/rsob.220224

**Published:** 2022-10-19

**Authors:** M. Fethullah Simsek, Ertuğrul M. Özbudak

**Affiliations:** ^1^ Division of Developmental Biology, Cincinnati Children's Hospital Medical Center, Cincinnati, OH 45229, USA; ^2^ Department of Pediatrics, University of Cincinnati College of Medicine, Cincinnati, OH 45229, USA

**Keywords:** morphogen gradient, clock, pattern formation, signalling, diffusion, fold change

## Abstract

Metazoan embryos develop from a single cell into three-dimensional structured organisms while groups of genetically identical cells attain specialized identities. Cells of the developing embryo both create and accurately interpret morphogen gradients to determine their positions and make specific decisions in response. Here, we first cover intellectual roots of morphogen and positional information concepts. Focusing on animal embryos, we then provide a review of current understanding on how morphogen gradients are established and how their spans are controlled. Lastly, we cover how gradients evolve in time and space during development, and how they encode information to control patterning. In sum, we provide a list of patterning principles for morphogen gradients and review recent advances in quantitative methodologies elucidating information provided by morphogens.

## Graphical abstract

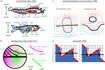

## 1. Cartesian coordinates of an embryo

**Figure 1.**
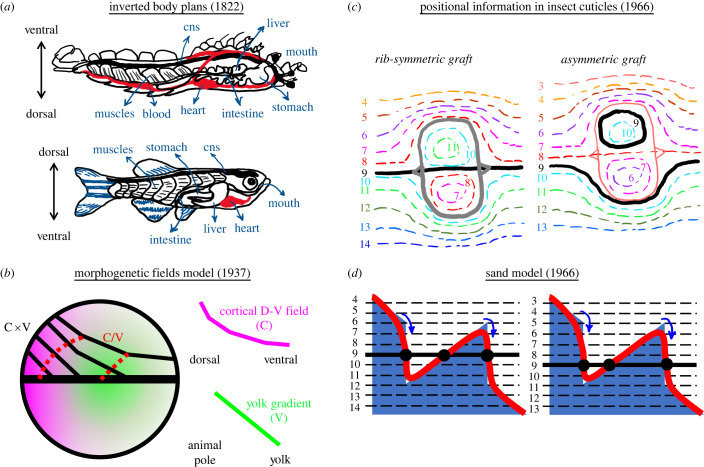
History of positional information before Wolpert. (*a*) Upside down (ventral is top) anatomy of crayfish, as an invertebrate model, adapted from Geoffroy St. Hilaire's 1822 work resembles dorsoventral axis patterning of vertebrates (shown here with zebrafish). The central nervous system (cns), muscles, liver, stomach and heart are formed from D-V order in zebrafish, whereas V-D in crayfish. (*b*) Dalcq and Pasteels model (adapted from [2]) for *Xenopus* blastula patterning under the influence of cortical dorsoventral field (C, magenta) and animal pole to yolk gradient (V, green). Product of C and V (black solid lines) together with the ratio between C and V (red dashed lines) splits blastula into morphogenetic fields. (*c*) Stumpf's experiments dissecting insect ectoderm and rotating 180° before grafting back shows a gradient encoded positional information (adapted from [3]). Solid oval lines are the borders of grafts. Colour-coded dashed isomixes following hair growth direction in the adult indicate positional identities. Thick black lines are rib formation corresponding to positional identity no. 9. Symmetric rib formation for symmetrically positioned grafting (left) fails when the graft domain is shifted slightly up (right). (*d*) Lawrence's ‘sand model’ to explain positional information for insect ectoderm experiments described in (*c*). Blue sand hill has an optimal slope providing positional information along the tissue. While gravity (i.e. diffusion in the tissue) tries to flatten the hill, sand friction (i.e. active transport) resists and reinforces the slope at an optimal level. Blue arrows indicate cells responding to drastic slope changes after grafting resulting solid red surface to form. Black dots highlight rib position for symmetric (left) and asymmetric (right) grafts.

Development of various metazoan embryos follows a common temporal and spatial pattern, as observed as early as 400 BC for birds and fish species [1]. This precise and reproducible nature of development requires cells to have a sense of space within the embryo. The first evidence for a coordinate system (embryonic body axis) existing within embryos can be dated back to 1822 when zoologist Geoffroy Saint-Hilaire realized dorsal–ventral inversion of body plans between vertebrates and arthropods (figure 1*a*) [4,5]. With the birth of experimental embryology at the dawn of twentieth century, Boveri [6], Driesch [7], Morgan [8], Spemann [9] and others perturbed positional information within developing blastula through displacement, dissection or unification of embryonic tissues. Embryologists realized a developing embryo split into two could successfully develop into two small organisms (Driesch, 1891, sea urchins, and 1895, starfish; Zoja, 1895, jellyfish; Crampton, 1897, tunicates; Wilson, 1893, amphioxus; Morgan, 1895, killifish as accounted for in [8]; and significantly pre-dating them: Haeckel, 1869, siphonophores [10,11]). Spemann's newt studies showed the dorsal axis must remain intact for this embryonic self-organization [9]. Embryo unification experiments performed in early 1900s revealed cartesian axes established within embryos: only parallelly aligned blastulae successfully morphed large size union embryos [12].

## 2. Fields and gradients

Classical embryology experiments led to ‘totipotent’ description of embryonic cells forming ‘self-regulating fields'. Transplantation experiments of Harrison in 1918 with ‘forelimb field’ [13] triggered identification of spatially distinct ‘fields’ within an embryo. Harrison's limb bud graft experiments (1921–1925) importantly revealed a time-ordered induction of orthogonal anteroposterior, proximodistal and dorsoventral axes patterning the limb bud [14]. In 1924, Hilde Mangold, under the supervision of Hans Spemann, used two distinguishably coloured newt species as host and donor and induced a secondary embryonic axis in the recipient newt embryo by grafting the upper blastopore lip region of the donor [15]. That region, named the organizer, was able to induce new axial identities (positional values) for the host cells in their vicinity.

In the meantime, Child observed a graded response of embryonic tissue to environmental factors [16,17] supporting existence of ‘axial gradient fields' within an embryo. However, those gradient fields were not generating patterns, instead they were graded enzymatic activities caused by a pattern asymmetry (polarity) existing within an embryo [18]. In 1929, Runnström developed double (antagonistic) gradient theory for animal-vegetative axis patterning of sea-urchin embryos; this theory was further experimentally detailed by his student Hörstadius [19]. In 1937, Dalcq and Pasteels merged ‘axial gradients’ idea with the long-acknowledged ‘organizer fields’ as ‘morphogenetic fields’. They proposed threshold ratios of two morphogenetic fields are the formative cause of embryonic cell fates [20]. A comprehensive review of these initial ‘threshold gradient’ ideas is provided by Gilbert [21]. The perpendicular ‘morphogenetic fields' theory of Dalcq-Pasteels attributed to gradients, for the first time, a ‘positional information’ beyond polarity (figure 1*b*).

## 3. Form-giving substances or morphogens

Late-nineteenth-century studies extending over a broad variety of species had convinced Driesch and Morgan that certain forming factors were ‘localized’ within the protoplasm of embryos [8]. Localized head-forming and tail-forming factors were already proposed in 1745 by Bonnet in regenerating adult Lumbriculus worms, as substances travelling towards and accumulating at opposing ends of animal fragments, hence creating polarity, to activate either head or tail ‘germs' (i.e. stem cells) [2,22]. Driesch further speculated on factors that determine the axial relations of the embryo and stated ‘prospective value of a blastomere is a function of its position’ [23]. Following Mangold and Spemann's discovery of ‘organizer’, Holtfreter and others showed even a dead organizer maintained its inductive capacity in grafts. These experiments provided first evidence for axial induction by hypothetical diffusive chemical substances [24]. To explain the ‘organizing’ capacity of certain ‘embryonic fields’, Waddington and colleagues proposed two types of chemical substances: ‘evocators’ and retrospective ‘individuators’, which act non-cell-autonomously [25]. The evocators would exist in a masked form throughout the embryo but are locally liberated or activated at organizers [26]. Inspired from ‘evocators’, in 1952, Turing coined the term ‘morphogen’ as ‘form producer’ substances chemically reacting within a tissue as they diffuse through it. Turing proposed concentration fluctuations for such diffusive substances could eventually lead to stable, periodic tissue patterns, the wavelength of which emerges from reaction rates and diffusion coefficient of morphogens [27]. However, Turing's ‘reaction–diffusion’-based patterning initially did not gain much appreciation among biologists. Waddington and Deuchar, the next year, disfavoured a Turing-type patterning mechanism in the context of somite segmentation problem: ‘It appears rather unsatisfactory to appeal to such an inherently chancy mechanism as this to explain a regular and basic phenomenon of development such as meristic segmentation’ [28].

## 4. Positional information

First experiments investigating positional values along an axis, beyond the mere presence of polarity, came in 1960s. Scientists used insect ectoderm giving rise to cuticles. Cleverly rotating, swapping and patching ectoderm tissues, Stumpf [29] and others [30] definitively showed existence of positional values along a gradient locally maintained by the cells (figure 1*c*). Ectoderm cells committed into certain cuticle identities and determined hair growth direction according to a hypothetical positional information. Stumpf interpreted these results as a diffusive substance establishing the gradient which provides their positional identity to the cells [3]. However, it was unclear to other scientists how a diffusive chemical from a source can establish ‘locally stable’ gradients [31]. Criticizing explanatory power of Stumpf's ‘diffusion from a source’ idea, Lawrence proposed the ‘sand model’ which further incorporates two additional mechanisms: 1- an awareness of offset from neighbouring values (i.e. slope), and 2- a ‘resistance’ to diffusion such as an uphill active transport which reinforce the slope of the gradient (figure 1*d*) [32]. Previously, Rashevsky had proposed that a less diffusive substance inhibiting the production of a faster diffusing metabolite and being displaced by it can establish polarized gradients by enhancing initial concentration inhomogeneities [33]. He further theorized concentration thresholds of that metabolite gradient can give rise to certain cell fate commitments (i.e. bone, muscle and skin of a limb). Self-enhancing reactions of one's own fates and repression of successive ones would maintain relative spatial positions of these commitments in a growing tissue, and successively pattern all three fates [34,35].

In 1968, Lewis Wolpert put flesh on the bones of under-appreciated theories and observations on ‘positional information’ as ‘the French Flag Problem’. Articulating on earlier experiments indicating positional values in embryonic tissues and spatial scalability of organ development, Wolpert proposed that a diffusive morphogen produced at a source and degraded at a sink, localized at opposite ends of tissue, can establish a linear gradient throughout it [36]. Threshold values of that morphogen can provide ‘positional information’ for multiple differentiation fates; e.g. high threshold and low threshold targets of the morphogen can divide the tissue into three zones, like three distinct colours of a French flag. Francis Crick mathematically estimated a diffusion coefficient for a hypothetical morphogen molecule and proposed how diffusion can readily establish these ‘source and sink’ gradients spanning some 50–100 cells (less than 1 mm) along the tissue within couple hours, parallel to experimental observations [18]. Wolpert's crystalline idea of a localized morphogen sink and local thresholds dictating various cell fates, popularized later as ‘the French Flag Model’ [37], gained thrust to explain ‘positional information’ within embryonic tissues (will be discussed in detail later).

The triumph of molecular genetics and its application to development since late 1980s provided compelling evidence for two facts: (1) A handful of signalling pathways are frequently used by cells for early developmental patterning [38]. (2) Many of these pathways (such as canonical Wnt, receptor tyrosine kinase (e.g. Fgf, Egf), receptor serine-threonine kinase (TGF-β superfamily: Nodal/Activin and BMP), retinoic acid, RA and sonic hedgehog, Shh) have secreted ligands and they establish gradients [39]. However, it is still debated what features of morphogen gradients (e.g. threshold, slope, temporal change, persistence) provide the ‘positional information’ for cells to organize and differentiate. Addressing this question demands us to first cover how the morphogen gradients are established and shaped.

## 5. What morphs a morphogen gradient?

**Figure 2.**
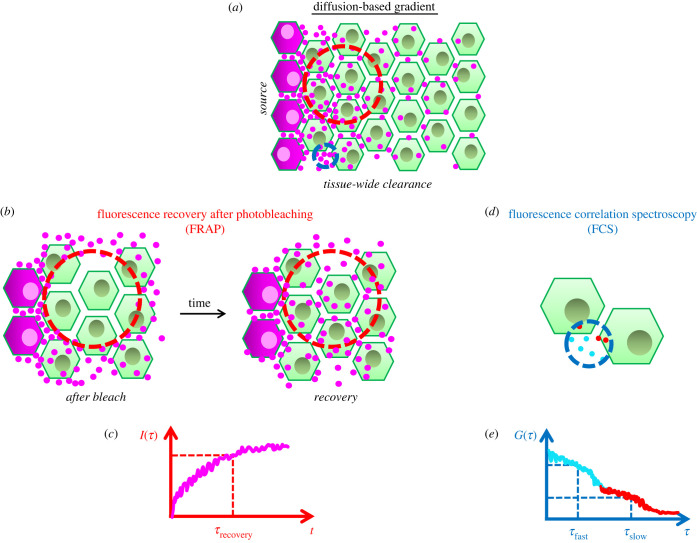
Diffusion-based gradient. (*a*) A gradient based on morphogen (magenta dots) diffusion is established over a 2-D tissue of cells (green). Morphogen is synthesized locally at magenta cells to the left and cleared tissue wide. Cell-internalized morphogen gradient shadows the external diffusive gradient. Regions of FRAP and FCS measurements are highlighted in red and blue dashed circles. Please note, a generic cellular sketch with extracellular regions is drawn here. However, the Bicoid gradient in Drosophila blastoderm is established in a syncytium of nuclei sharing the same cytoplasm. (*b*) FRAP measurements bleach out fluorescent morphogens from a large region (red dashed circle covering cells and extracellular space) and observe fluorescence recovery over time. (*c*) Fluorescence recovery time scale (*τ*_recovery_) depends on endocytosis, degradation, recycling, etc. dynamics besides the morphogen diffusion in extracellular space. (*d*) FCS measurements focus on a narrow extracellular spot (blue dashed circle next to cell membranes, size is exaggerated) correlating the fluorescence signal over time as molecules diffuse in and out of focal volume. (*e*) Signal's autocorrelation function (ACF) decays over longer lag times (*τ*) depending on the average diffusion speed of fast (cyan) and slow (red) populations. Decay amplitudes of ACF provide percentages of two populations.

How a gradient is established eventually determines the spatio-temporal evolution of its shape. Establishment of a gradient through diffusion requires three steps: 1- localized synthesis, 2- diffusion and 3- clearing of the morphogens. A localized sink for clearing [18] would form a gradient with a linear slope (Source and Sink Model). However, if the clearance (by degradation, immobilization or leakage) happens throughout the tissue, the gradient decays exponentially [40] (figure 2*a*). First discovered morphogen, exponential Bicoid gradient patterning anterior axis and positioning cephalic furrow in Drosophila [41] indicated tissue-wide clearance. Soon after fertilization, Bicoid proteins are locally synthesized from maternally deposited mRNAs at the anterior end of Drosophila embryo [42] and diffuse to establish an anteroposterior exponential gradient. The diffusion speed of Bicoid was first measured with fluorescence recovery after photobleaching (FRAP) (figure 2*b,c*) as 0.3 µm^2^ s^−1^ [43], an order of magnitude lower than similarly sized inert molecules diffusing within the same tissue. This result triggered searches for alternative hypotheses to explain the gradient establishment [44]. However, later measurements using fluorescence correlation spectroscopy (FCS, [45,46]) (figure 2*d,e*) and tandem florescent timer system [47] pinned Bicoid diffusion speed to approximately 7 µm^2^ s^−1^ for freely diffusing internuclear portion, or 2.9–4.9 µm^2^ s^−1^ range, incorporating nuclear trapping effects. In addition, the amplitude of Bicoid gradient increases for several nuclear cycles, briefly reaches a quasi-steady state and diminishes at the onset of gastrulation [48,49]. The diffusion-based model appears to explain these spatio-temporal dynamics of the Bicoid gradient the best [46,47].

Numerous morphogen gradients in various organisms and tissues have been identified following the discovery of Bicoid gradient. A diffusion-based mechanism was also shown to be instrumental for establishing many such gradients: e.g. Gurken (a TGF-α like ligand)—EGFR signalling gradient patterning dorsoventral axis of Drosophila oocyte [50], Wingless (Wnt5 homologue) gradient patterning Drosophila wing disc dorsoventrally [51,52], Fgf signalling discriminating mesoderm from endoderm during vertebrate gastrulation [53], Sonic hedgehog (Shh) patterning vertebrate limb anteroposterior axis [54] and neural tube ventral axis [55], Nodal signalling inducing mesendoderm [56–58] and left-right asymmetry [59], and BMP gradient patterning dorsoventral axis in vertebrate gastrula [60–62].

Clearly, source and sink mechanism does not parsimoniously account for the establishment of morphogen gradients in various organisms and tissues. A major concern raises from the very nature of diffusive random walk: Duration of spread via free diffusion increases with distance squared [63]. Diffusion-based explanations are limited to gradient ranges shorter than hundreds of microns for biologically relevant time scales (several hours) [18]. Indeed, many morphogen gradients are established within several hours of embryonic development [48,56,62,64]. However, this criterion is valid if the source and sink morphing the gradient are perfectly localized at far ends and a steady-state gradient is required to provide any positional information. But, as mentioned previously, clearance of morphogens happens throughout the tissue. Moreover, sources of long-range gradients are also usually dispersed. For instance, graded expression of *bmp* [65] and *nodal* ligand transcripts in vertebrate blastula and the *bicoid* RNA gradient in Drosophila blastoderm [66] span about a third or more of total signal gradient range. Lastly, well-studied gradients such as Bicoid in blastoderm [48,67,68] and Dpp in wing disc [48,67–72] provide patterning information to cells on the go (without reaching a steady state).

A significant case for such long-range morphogen signalling is Fgf gradient established in presomitic mesoderm (PSM) of vertebrates, controlling sizes of somites. As the tail axis of the embryo elongates, groups of cells join tailbud while they also transcribe *fgf* RNA. Continuing axis elongation eventually displaces those cells far anterior from the tailbud which stops the *fgf* transcription. In result, embryos display *fgf* RNA gradient decreasing from posterior to anterior PSM. This RNA gradient is expected to be translated into a Fgf ligand gradient [73]. Conversion of a transcriptionally active signalling source (tailbud) into a graded RNA source can maintain the Fgf gradient along the PSM as long as greater than 1.5 mm in mouse and snake [74]. Cells at a specific mid-PSM location commit to mark somite boundaries [75–77]. This positional information is provided to cells by the Fgf morphogen gradient, as it retreats over mid-PSM cells posteriorly [78]. Notably, even though described RNA gradient mechanism does not require any ligand diffusion to establish a morphogen gradient, tissue manipulations in three-dimensional zebrafish PSM explants argue for the importance of ligand diffusion: 1-) Physical arrest of axis elongation in PSM explants results immediate determination of smaller somites in mid-PSM. 2-) Removal of *fgf* transcription source (tailbud) also reduces somite sizes immediately at a distance greater than 300 µm from the source in mid-PSM [78]. These results were explainable in a model only when Fgf ligands diffused swiftly in extracellular space over several PSM cells per minute [78], comparable with FCS diffusion measurements for Fgf8 in zebrafish blastula [53].

The extracellular diffusion-based mechanism was strongly disputed for the Decapentaplegic (Dpp, BMP homologue) gradient controlling the growth and patterning of the A-P axis of *Drosophila* wing imaginal disc, a two-dimensional epithelium tissue. Dpp is locally expressed in a thin dorsoventral line of cells separating anterior and posterior compartments of the wing disc and establish an exponential gradient increasing in range as the wing disc grows. In 2000, researchers raised several concerns against the diffusion model by observing fluorescently tagged Dpp molecules: 1- Most of the Dpp molecules were residing within the cells instead of diffusing along the extracellular matrix. 2- Time controlled and local inhibition of Dynamin-mediated endocytosis created immediate restrictions in Dpp gradient profile and caused the morphogens to get stuck in the clone border [79]. These observations together with low apparent diffusivity of Dpp morphogens measured by FRAP experiments (*D*_app_ = 0.1 µm^2^ s^−1^) were interpreted in favour of transcytosis model [80] (endocytosis-driven transport of morphogens from one cell to the next). Furthermore, theoretical arguments were raised against the extracellular diffusion model by highlighting how receptors could trap morphogens and prevent gradient establishment [81].

**Figure 3.**
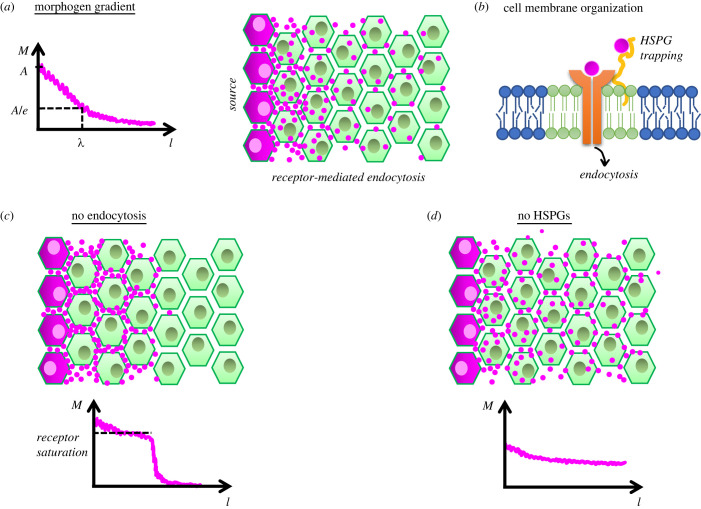
How to establish an informative morphogen gradient. (*a*) Morphogens diffusing from a localized source (magenta) establish an exponentially decaying gradient over tissue space (*l*). A characteristic decay length (*λ*) can be defined as where the gradient amplitude (*A*) drops to its one by *e*^th^ value. (*b*) Cell membrane is spatio-temporally organized as saturated lipids (green) phase separate into liquid-ordered nanodomains (i.e. rafts) in between unsaturated lipids (blue). Certain HSPGs, such as glypicans (yellow) associate with ordered nanodomains trapping morphogens and providing them to receptors (orange). Ligand-bound receptors undergo endocytosis. (*c*) Endocytic block results trapping of morphogens in extracellular space by receptors until reaching receptor saturation levels. This prohibits establishment of a gradient and diffusion of morphogens beyond a space. (*d*) Removal/absence of HSPGs results gradients to become shallow and unable to provide a positional information.

Contrary to these objections, later studies showed that ligand-binding induced clearance of receptors (endocytic recycling or degradation) is actually essential to establish a morphogen field through extracellular diffusion [72,82] (figure 3*a–c*). Informative (neither too steep nor too shallow) gradients can be established via ligand diffusion over dozens of cells within several hours if (1) cell surface receptor density does not exceed a certain level (in the order of thousands per cell), (2) the ligand-bound receptors are cleared off from the cell surface, and (3) ligands have slow association kinetics (*K*_a_ ∼ 10^4^–10^5^ M^−1^ s^−1^) with their receptors [82]. These three conditions appear to be met for quantitatively investigated morphogens [83,84], pointing to optimality of ligand-receptor systems used as morphogens in nature. Such optimality provides morphogens an opportunity to establish meaningful gradients for a broad range of receptor occupancy fractions (from approx. 0.1 up to less than 0.8) [82]. Most of the morphogen population can get bound to receptors and internalized within cells as long as the remaining extracellular portion keep diffusing freely (i.e. with high speeds in majority) to sustain the gradient. As such, internalized receptor-bound morphogens shadow the shape of extracellular gradient [82,85].

### 5.1. Morphogen principles – general background

— A handful of cell signalling pathways are facilitated throughout the development for patterning various tissues.
— Many signalling pathways display graded activation with highly diffusive activators, ligands and/or inhibitors.
— Beyond a certain size of tissue and within developmental time scales, diffusion is not an effective method to establish gradients from a localized source. Sources of long-range gradients are usually dispersed. However, ligand diffusivity remains instructive even within long-range gradients.
— In ligand-receptor systems, spatially meaningful morphogen gradients are established upon an intricate balance of cell surface receptor occupancy via receptor clearance and slow association kinetics.

## 6. Control of a morphogen gradient's diffusive span

Similar to Bicoid, Dpp morphogens were measured to diffuse at microscopic scale fast enough to form a gradient along the wing disc: approximately 65% of the population at cell–cell junctions diffuse with 21 µm^2^ s^−1^ speed [64]. The mismatch between FRAP and other more localized correlation microscopy techniques stirred extensive debates on which measurement matters most for the cells [86]. Tissue packing (restricted three-dimensional space for diffusive molecules) would lead to lower long-range *D*_app_ calculations from FRAP (or fluorescence spread after photoactivation, FSAP) regions as big as several hundred cells [56] (figure 2*b,c*) with respect to local *D*_app_ calculations from FCS focal volumes covering approximately 200–300 nm in diameter even for inert molecules like GFP [53,56] (figure 2*d,e*). However, the mismatch between FCS and FRAP measurements for discussed morphogens as well as other diffusive morphogens such as Bmp2 [62], Fgf8 and Nodal signalling activators Cyclops and Squint in zebrafish [44] is beyond what is explicable with tissue packing. Case for Nodal signalling is more complicated: although both activators and repressors of Nodal signalling locally diffuse at similar speeds [44], diffusion of activators but not repressors are significantly hindered in long range [56]. Recent work in zebrafish points to Nodal co-receptor One-eyed-pinhead (Oep) [87] for this hindrance [88]. Nodal gradients are robustly established owing repressive feedback from faster diffusing Lefty inhibitors in a Turing-type reaction–diffusion setting [56]. Interestingly, both mutants lacking Lefty inhibitors [89] and ligand trapping Oep co-receptor [88] result in pervasive Nodal signalling with expanded gradients and lethal phenotypes. Evidently, what sets up the final reach of a morphogen field depends on the regulation of long-range diffusivity and signalling of ligands [90].

Restriction of FRAP/FSAP measurements to smaller regions next to cell membranes closes the gap between FCS measurements [85]. Nevertheless, FRAP/FSAP measurements alone fail to untangle long-range diffusion from other artefacts: FRAP experiments photobleach fluorescently tagged ligands within a region, certain amount of which exist within cells, and waits for bleached ligands to be replaced by fluorescent ligands diffusing from outside of the region, binding to receptors and being internalized (figure 2*b*). The calculated time scale of that recovery depends on multiple factors as how tight cells are packed, receptor turnover/degradation dynamics besides the diffusivity of ligands (figure 2*c*). Moreover, both lipid modified (like Wnt and Hh) and water soluble (like Fgf and Bmp) morphogens alike interact with sticky molecules extracellularly presented by the cells, heparan sulfate proteoglycans (HSPGs), which trap ligand's long-range diffusivity. Inactivation of this trapping effect (i.e. via competitive binding by injected soluble heparins) increases long-range diffusivity of Fgf8 by about 10-fold and significantly decreases the mismatch between local versus global diffusion rates [44]. In microscopic scale, measured through FCS (figure 2*d*), perturbation of HSPG trapping alters the ratio of slow diffusing versus fast diffusing Fgf8 population [53] (figure 2*e*). Along these lines, only shed but not the membrane anchored form of glypicans (GPI-anchored HSPGs: Dally and Dally like, Dlp in Drosophila) increase the spans of Dpp, Wg and Hh [91–94] gradients in Drosophila wing disc. Hedgehogs and Wnt-family morphogens are post-translationally lipid modified: cholesterol moiety attachment to Hh [95] and palmitoylation of Wnt [95,96] are essential for their signalling. Gradients of those morphogens usually span shorter [51,97], in comparison with the gradients of soluble morphogens within same tissues [78,80,98].

Local diffusivity of Wnt ligands was measured with FCS in *Xenopus* gastrula; fast-diffusing fractions were also found to comprise majority (61–70%) of extracellular populations [85] and the presence of diffusive Wnt binding proteins (i.e. Frsp) increases Wnt gradient span [99]. However, lipid-modified morphogens would differ from soluble morphogens for their unique associations at cell surface. Cell membranes display structural heterogeneity and compartmentalize into cholesterol-stabilized lipid nanodomains (i.e. lipid rafts) [100] (figure 3*b*). These highly dynamic nanodomains, anchored by sub-membrane actin cytoskeleton [101], facilitate protein sorting [102] and enhance signalling fidelity [103]. Importantly, both the kinds of morphogen lipid-modifications (cholesterol-moiety and palmitoylation) selected in nature and the proteoglycan types they interact with on cell surface (GPI-anchored glypicans) indicate a preference for these ordered nanodomains [104] (figure 3*b*). Affirmatively, canonical Wnt signalling ligand-receptor binding in zebrafish is shown to occur preferentially in these nanodomains and domain disruption resulted reduced signalling [105]. Dimerization of glypicans or receptors upon ligand binding can further stabilize the lipid nanodomains, as shown for inert GPI-anchored fluorescent proteins [104], and enhance the cell's capacity to relay signalling robustly.

### 6.1. Morphogen principles for ligand-receptor systems I

— An activator–inhibitor system with various long-range diffusivities can establish a spatial domain for the signalling gradient (i.e. gradient span), even if the local microscopic diffusion coefficients are similar.
— Morphogens interact with co-receptors and extracellular sticky molecules (e.g. proteoglycans) presented by cells which hinder their long-range diffusivity.
— Lipid modifications of certain morphogens restrict their gradient span further than unmodified counterparts establishing gradients within same tissues.
— Both lipid-modifications and proteoglycans selected in nature indicate a preference for lipid-ordered nanodomains in cell membranes as signalling hubs. These nanodomains can enhance cell's capacity to relay the signal robustly.

## 7. Non-signalling coreceptors and gradient span

Recently, Stapornwongkul and colleagues rescued growth and patterning phenotypes of Dpp mutants by engineering a semi-synthetic GFP morphogen gradient [106]. This innovative work made a manifest case for source and sink gradient mechanism. For that purpose, Type-I and Type-II Dpp receptors (Tkv and Punt) were linked to high-affinity GFP antibodies recognizing two separate GFP epitopes to work as functional GFP receptors. Once GFP was synthesized locally at A-P boundary downstream of *dpp* promoter, these ubiquitously expressed receptors activated Dpp signalling (phosphorylated Mad, pMad) in the absence of Dpp. However, low copy number of receptors led to broad (not significantly graded) activation of pMad due to GFP leakage into the tissue space basal to wing disc. Although increasing receptors successfully restricted GFP within the wing disc and established a gradient, its span fell shorter than that of wild-type pMad gradient. However, introduction of GPI-anchored, non-signalling, low-affinity GFP receptors downstream of dally promoter generated pMad gradient similar in span to that in wild-type and rescued Dpp phenotypes [106].

By which mechanism do low affinity non-signalling receptors (such as glypicans) help to establish a gradient with proper range? (figure 3*d*) Why do those HSPGs interact with many ligand types (Shh, Wnt, TGF-β, Fgf) establishing gradients throughout metazoan? Previously, genetically mosaic patches of dally mutants in wild-type wing discs were shown to prohibit observation of Dpp-GFP molecules trespassing them [107]. These results were interpreted in favour of GPI-anchored proteoglycans being essential for expanding the span of Dpp gradient [86,108]. However, they can simply be attributed to internalization dynamics of Dpp. Glypicans are shown to facilitate receptor-binding and internalization of Dpp ligands (also see ‘lipid nanodomains’ discussion above). As discussed previously, majority of Dpp molecules exist within cells. Mosaics lacking dally are less effective for internalizing and stabilizing Dpp molecules and will appear as if prohibiting Dpp transfer [64]. A secreted molecule Pentagone (Pent), repressed by Dpp signalling, is expressed complementary to Dpp gradient in central wing pouch. *pent* mutation shrinks the Dpp gradient, and mutants are not able to sustain wing disc growth: Dpp gradient does not expand despite compromised tissue growth continues [109]. On the flip side, Dpp gradient span expands further when Pent is overexpressed [109]. How does Pent control the gradient span? Glypican biosynthesis is facilitated in lateral wing cells whereas Dpp signalling at the central region represses expression of both its receptor, Tkv [110], and non-signalling glypican, Dally [92]. In sum, Tkv and Dally exhibit an opposing gradient to Dpp signalling. A recent elegant study showed when Tkv and Dally are uniformly expressed, Dpp gradient fails to expand despite tissue growth; recapitulating *pent* mutant phenotype [69]. Moreover, reduction of HSPG function locally at *pent* expressing domain mimics *pent* overexpression and expands Dpp gradient; whereas tissue wide reduction shrinks pMad gradient similar to *pent* mutants [69]. Intriguingly, ubiquitous expression of GPI-anchored non-signalling receptors could not expand the span of semi-synthetic GFP signalling gradient like they did when expressed under dally promoter [106]. Although synthetic and minimal design of GFP source-sink gradient system eliminated many feedbacks discussed above [86], it is plausible that functional pMad signalling restricts non-signalling GFP receptor expression to lateral cells, where Pent can properly function for enhancing the span of the GFP gradient. Fittingly, ectopic expression of dally in posterior wing disc boosts pMad signalling locally and does not expand the gradient, when Pent is not present [109]. Pent, being settled at the correct location, appears to hold the control knob of gradient span by recycling of Dpp ligands [72] and clearance of Dally glypicans from cell surface.

Reasonably, one can ask why cells don't simply produce lower levels of those receptors at first instead of using the complicated regulation through Pent [69]. Molecular details remain unclear. In the light of synthetic GFP gradient experiments [106], it is tempting to speculate that cell surface proteoglycans are essential [111] to restrict dimensionality for diffusion [112]—similar to functionality of cytonemes [113]—for long-distance signalling; especially in two-dimensional leaky tissues such as wing disc. Live observations at the cell membrane level would be beneficial to solve the mystery of how GPI-anchored HSPGs appear to facilitate both gradient span and receptor-binding/ligand trapping. If GPI-anchored glypicans would facilitate morphogen field expansion with their fast membrane diffusion [106], it should have shown up in FCS measurements. Although diffusivity of GPI-anchored molecules is fast on cell surface [102,104], our previous calculations for inert GPI-anchored fluorescent proteins in mammalian cell culture estimated dimerization would lengthen average dwelling time of such receptors within lipid nanodomains five to ten-fold [114]. Single-focus FCS measurements provide an averaged-out diffusion coefficient from the observed focal volume (figure 2*e*) and fail to explain effects of *pent* [69] or *dally* [64] mutants. However, when paths of different molecules cross, they can slow each other down (steric interactions), prefer to spend time together (domain-association), or interrupt each other's paths (fence trapping / hop-diffusion) [44]. All those interactions would result in similar outcomes from a classical FCS experiment. It is possible to untangle different modes of diffusion by observing molecules over multiple volumes/areas [115]. Camera-based FCS imaging along a plane of illumination lets mining these dynamics from a simultaneous data by treating square-binned camera pixels as multiple observation areas [104] and enables quantification of time course changes for different modes of diffusion [101]. Application of such quantitative microscopy tools might help reaching the design principles for morphogen gradient span control.

### 7.1. Morphogen principles for ligand-receptor systems II

— Non-signalling coreceptors ubiquitously found in thin tissues, such as Drosophila wing discs, might help confine diffusivity of morphogen within the two-dimensional tissue space, prevent tissue leakage and expand the gradient span.
— A balance between the trapping effect and expansion of gradient span involves additional molecular players (e.g. Pent in wing disc, by controlling receptor clearance/recycling).

## 8. The scaling problem: spatiotemporal evolution of morphogen gradients

**Figure 4.**
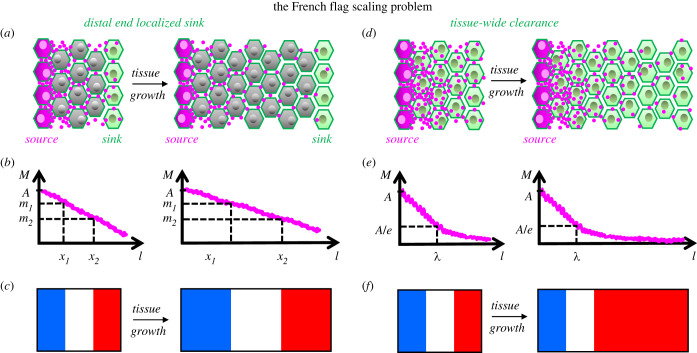
The French flag scaling problem. (*a*) For a hypothetical tissue growing in size (left to right) only distally localized sink cells (green) clear the morphogens from the tissue as they move away from the source (magenta). (*b*) Distally localized sink dilutes the morphogen gradient with amplitude *A* over the tissue length linearly. Threshold morphogen values *m_1_* and *m_2_* specify positions (*x_1_* and *x_2_*) further away from each other with growing tissue size. (*c*) Linear gradient maintains scaling invariance of three coloured domains of a ‘French flag’. (*d*) Cells throughout the tissue endocytose and clear the morphogens. (*e*) Tissue-wide clearance and uniform diffusion results an exponential morphogen gradient with a characteristic decay length, *λ*, independent of tissue size. (*f*) Scale-invariant patterning fails for an exponential gradient.

If a morphogen gradient provides positional information at different threshold concentrations at several locations in a tissue whose size varies during development, then the gradient has to be scaled with tissue size (i.e. the scaling problem; figure 4). Wolpert's positional information model takes care of this issue assuming a linear morphogen gradient with distally localized source and sink (figure 4*a–c*). Linearity of the gradient was instrumental in Wolpert's model to maintain proportional positional values dictated by local thresholds, as the source-sink gradient scales with growing tissue [116]. However, an exponential (nonlinear) morphogen gradient [40] challenges that model as it fails to scale with tissue size [117]: The characteristic length for exponential morphogen field, *λ*, increases with diffusion coefficient and decreases with clearance rate, however, does not depend on amplitude (*C*_0_) and the span (*L*) of the gradient (see box 1, equations (8.1) and (8.2), and figure 4*d,e*). Therefore, threshold readouts of that gradient will not maintain scale-invariant positional information (figure 4*f*). In turn, either diffusion speed or clearance rate should be modified as the tissue size alters, or the morphogen flux (production rates in source) should be precisely updated for scaling [119].

Box 1. **A boundary value problem of morphogen gradients.**

One can define concentration (*C*) dynamics of a morphogen synthesized within a production zone at origin (*x* = 0 − *x*_0_) and diffusing along and cleared within a simplified 1 − D tissue of length *L*, as follows:

8.1 $$\frac{\partial C}{\partial t}=k_{p}{|}_{0}^{x_{0}}+D\frac{\partial^{2}C}{\partial x^{2}}-Ck_{c}{|}_{0}^{L},$$

where *k*_p_ and *k*_c_ are rate constants for production and clearance and *D* is the diffusion coefficient of the morphogen. Assuming closed boundary conditions (∂*C*/∂*x*|*_x_*_=0_ = ∂*C*/∂*x*|*_x_*_=_*_L_* = 0), this will result in a steady-state (*∂C*/*∂t* = 0) solution as a gradient of amplitude *C*_0_ = *C*_1_ + *C*_2_ between *x* = 0 − *x*_0_, and sum of two exponential functions beyond:

8.2 $$C{|}_{x_{0}}^{L}=C_{1}e^{-(x-x_{0})/\lambda }+C_{2}e^{(x-x_{0})/\lambda },$$

with characteristic length, $\lambda =\sqrt{D/k_{c}}$ (figure 3*a*). Considering the boundary condition at *x* = *L* one can also relate two amplitudes of gradient terms *C*_1_ and *C*_2_ as follows: $\partial C/\partial x{|}_{x=L}=0=\lambda (-C_{1}e^{-(L-x_{0})/\lambda }+C_{2}e^{(L-x_{0})/\lambda })$, so $C_{2}=C_{1}e^{-2(L-x_{0})/\lambda }$ [118]. A basic assumption we made here is that there is no advection of morphogens.

We can now attempt to solve the previous equations for gradient amplitude, *C*_0_. Total morphogen levels existing along the tissue at steady state can be written by integrating the concentration equation above over the tissue length:

8.3 $$\begin{array}{rl} M & =C_{1}(1+e^{-2(L-x_{0})/\lambda })x_{0}+C_{1}\int_{x_{0}}^{L}\left(e^{-(x-x_{0})/\lambda }+e^{(x+x_{0}-2L)/\lambda }\right)\;dx\\ & =C_{1}(x_{0}(1+e^{-2(L-x_{0})/\lambda })+\lambda (1-e^{-2(L-x_{0})/\lambda })). \end{array}$$

Note that we here simplified the Boltzmann sigmoid function within the production zone into a linear average. Tissue size extremities (which we will discuss from equations (8.7)–(8.10)) hold true with or without this simplification. Those *M* molecules of morphogen are produced over a distance of *x*_0_ and degraded throughout the tissue of length (closed boundary conditions). Therefore, *k_c_M* = *k_p_x*_0_ is in steady state. So, one can solve for the gradient amplitude as follows:

8.4 $$C_{0}=C_{1}(1+e^{-2(L-x_{0})/\lambda })=\frac{k_{p}}{k_{c}}\cdot \frac{x_{0}(1+e^{-2(L-x_{0})/\lambda })}{x_{0}(1+e^{-2(L-x_{0})/\lambda })+\lambda (1-e^{-2(L-x_{0})/\lambda })}.$$

In result, we can express morphogen levels at a given position *x* along the gradient as follows:

8.5 $$C(x)=\frac{k_{p}}{k_{c}}\cdot \frac{x_{0}e^{-(x-x_{0}/\lambda )}(1+e^{2(x-L)/\lambda })}{x_{0}(1+e^{-2(L-x_{0})/\lambda })+\lambda (1-e^{-2(L-x_{0})/\lambda })}.$$

We can easily put this morphogen concentration function in the form of

8.6 $$C(x)=\frac{k_{p}}{k_{c}}\cdot \frac{x_{0}\cos h(L-x/\lambda )}{x_{0}\cos h(L-x_{0}/\lambda )+\lambda \sin h(L-x_{0}/\lambda )},$$

in which cosh(*α*) = (*e^α^* + *e^-α^*/2) and sinh(*α*) = (*e^α^* − *e^−α^*/2) is a hyperbolic trigonometric function.

To address this potential scaling problem, Barkai group proposed a model [120] where expression of a diffusive and long-lived ‘expander’ molecule is repressed by the morphogen signalling. This expander, in turn, is defined to facilitate morphogen's spread by reducing its trapping (by receptors etc.) or degradation. Expander will be highly expressed opposite to the morphogen gradient and will diffuse and invade the morphogen field. This feedback to source will then expand the morphogen field to the extent it will eventually shut off the expander's production (tissue size sensing). Expansion-repression model would keep morphogen levels stable at the distal end (enough to suppress expander production) and crudely maintain proportions of positional information for similar thresholds [120]. Pent-secreted molecule, discussed above, was proposed to be the ‘expander’ for Dpp signalling in wing imaginal disc [121] due to its three features: (1) expression of *pent* is repressed by Dpp signalling [109]; (2) scaling of Dpp gradient fails in *pent* mutants [122]; and (3) Pent is reducing morphogen trapping, so facilitating diffusion by lowering receptor activity [109]. However, diffusivity of Pent molecules beyond a narrow range (approx. 5–10 µm) is disputed [69,72]. Furthermore, local expression of receptors within *pent* expression domain recapitulated *pent* mutant phenotype [69]. These findings might indicate Pent lowers receptor activity locally within its expression domain but does not function as a long-range expander as proposed by the expansion-repression model [69]. In other tissue settings, a flip-view alternative to expansion-repression model (induction-contraction) can work similarly to facilitate gradient scaling by pinning morphogen value at the distal end [123,124].

Alternatively, changes in the production zone can also provide approximate scaling for morphogen gradients under certain conditions: Solution for the morphogen concentration (box 1 equations (8.5) and (8.6) provides certain insights about how similar values of the morphogen can correspond to similar relative positions for a tissue of changing size, i.e. *scaling*. For large tissues with small sources of morphogens (*x*_0_ ≪ *λ* ≪ *L*):

8.7 $$x_{0}(1+e^{-(2(L-x_{0})/\lambda )})\ll \lambda (1-e^{-(2(L-x_{0})/\lambda )})\approx \lambda .$$

In other words, cosh((*L* − *x*_0_)/*λ*) ≈ sinh((*L* − *x*_0_)/*λ*). So,

8.8 $$C∼\frac{k_{p}}{k_{c}}\cdot \frac{x_{0}}{\lambda }\cdot e^{-\frac{x-x_{0}}{\lambda }},$$

meaning, if the production zone (*x*_0_) increases exponentially with tissue growth, for a position *x* − *x*_0_ = *p*(*L* − *x*_0_), 0 ≤ *p* ≤ 1 away from the source, morphogen concentration will not vary for *p* scaling factor, despite characteristic decay length, *λ*, being constant. For instance, if the tissue length beyond the source (*L* − *x*_0_) doubles, $e^{-(p(L-x_{0})/\lambda )}$ term for *p* scale-invariant position along the tissue will decrease by 1/*e*^2^ which can be compensated by *e*^2^-fold exponential increase of production zone.

At its face, exponential regulation of production zone with tissue size might look far fetching. We will try to alleviate this concern, by focusing on an interesting scaling example. Influential work by J. Cooke in 1975 showed individual somites scale in size with body length, using size-reduced Xenopus embryos [125]. Interestingly, for tail somites of three vertebrate species (i.e. zebrafish, chick and mice), size of a somite is also scaling with the size of available PSM tissue at the time of boundary determination [78]. Fgf gradient provides positional information for somite boundaries in the PSM. Scaling automatically comes from how the gradient is laid out: Tail elongates with a more-or-less steady pace [78]. *fgf8* is only transcribed in cells located in the tail bud and it is no longer transcribed in cells displaced from tail bud into PSM due to axis elongation. That lays out an exponential RNA gradient along the PSM tissue [73]. Characteristic decay length of the RNA gradient depends merely on how fast RNA is degraded (no diffusion term). In zebrafish, the PSM continually decrease in size over somitogenesis, following a brief steady profile [74]. Due to its fast degradation, *fgf8* gradient does not span the full size of large PSM at early somite stages. Although the PSM shrinks, *fgf8* gradient shape does not change until about mid-somitogenesis (approx. 14 somite stage) [78]. Likewise, downstream Fgf signalling gradient (read out by ppERK effector molecule levels) does not scale for those early stages and scaling of somite sizes with the PSM sizes is also not observed [78]. At later stages, the PSM size becomes comparable with the RNA gradient range and starts shrinking the *fgf8* gradient [78] due to an unknown mechanism. Thereafter, the range of exponential *fgf8* gradient decreases proportionally with PSM tissue size. This exponential reduction in source can intuitively explain (equation (8.8)) scaling of Fgf signalling gradient for tail somite stages and consequential scaling of the somite sizes [78].

Although we performed calculations for an extremely simplistic geometry, many diffusive morphogens such as BMP, Nodal and Fgf signalling of vertebrate blastula or Dorsal/Dpp signalling of Drosophila blastoderm pattern over spherical geometries. Yet, similar arguments are still valid. Due to Laplacian operators in spherical coordinates, a diffusive morphogen emanating from a local source and degraded throughout a spherical surface establish a gradient dependent on the radius of embryo; so does not maintain a scaling for size variations observed between embryos as detailed in Weyer *et al*. [126]. This 1977 study modelling Xenopus mesoderm induction proposed either source amplitude to be controlled with embryo sizes, for scale invariance, or sink to be effectively localized to distal end. Indeed, highly diffusive Nodal inhibitor Lefty is shown to sense the size of embryo by accumulating higher at smaller embryos, hence reducing the amplitude and range of Nodal gradient [127]. Similarly, selectively bred wild-type Drosophila eggs with bigger sizes attain scaled patterning by having volume-proportional deposition of *bicoid* RNA, i.e. source amplitude [128]. A more recent Drosophila study leveraged maternal shRNA knockdown of atypical cadherin *fat2*, a core planar cell polarity gene, specifically in oocytes to end up with shorter A-P (approx. 15%), wider D-V (approx. %4) length *fat2RNAi* embryos, overall, slightly smaller (%8) than the wild-type volumes. Supportively, *fat2RNAi* embryos displayed wild-type-like levels of *bcd* RNA, resulting in a less-slanted Bcd morphogen gradient for short A-P axis embryos. Those observations further emphasize the tissue geometry for scaling phenomena.

On the opposite end of scale in box 1, equation (8.5), when the tissue is drastically small:

8.9 $$\frac{L-x_{0}}{\lambda }\ll 1;\;\mathrm{then},\;(1-e^{-(2(L-x_{0})/\lambda )})\approx \frac{2(L-x_{0})}{\lambda },$$

and we can rewrite concentration as:

8.10 $$C\approx \frac{k_{s}}{k_{c}}\cdot \frac{x_{0}}{L}\cdot \left(1-\frac{x-x_{0}}{\lambda }\right).$$

This linear relationship resembles Wolpert's localized source and sink model. However, in the latter, sink is localized; degradation is unaltered regardless of how far sink moves away from the source. So, the concentration at neither the source nor the sink changes, tissue expansion only alters the slope and scaling happens automatically. On the other hand, in the case of equation (8.10), bigger tissue size equates to more degradation per unit time, so the production should keep pace with it. In result, the production zone should increase linearly with tissue growth for scaling to work.

Dpp gradient of Drosophila wing disc appears to start scaling this way when the posterior disc zone (tissue of the gradient) is comparable in size with the characteristic decay length (approx. 10 µm) of Dpp signalling (pMad) gradient and keeps scaling until about posterior tissue length reaches approximately 100 µm over a course of approximately 2 days [69,72]. Similar to somitogenesis, gradient scaling is transient in wing disc as the tissue keeps growing for more than 4 days [72,129]. Opposite to the case for PSM size during somitogenesis, wing disc size increases over time, as the growth is controlled by the Dpp gradient [70,130]. Also, unlike Fgf gradient of PSM tissue, source of Dpp protein gradient is strictly localized by Shh signalling within a narrow margin between anterior and posterior disc zones [130]. Using biologically functional Dpp–GFP molecules, Wartlick *et al*. measured this source to grow approximately linearly in size with the growing tissue [129]. The gradients of Dpp–GFP ligand [129], signal transducer pMad [122], and the negative feedback regulator Dad [122,129] all increase in amplitude over time and exhibit size scaling. Although those observations fall in line with back of envelope calculations presented above for small tissues, they fail to maintain scaling beyond a size as observed in *pent* mutants [122]. Dpp gradient in wing disc facilitates further scaling up until third larval instar stage [69] by repressing its receptor expression [110] and through Pent's reduction of co-receptor (HSPGs) activity [109] and facilitation of ligand recycling [72]. These regulations restrict effective sink position further away from the source and the system verges on a localized source and sink model [69]. Effective localization of sink activity to the distal end through such receptor modulation [131] or ligand inactivation [61] might be a principal mechanism for large range gradients. In Xenopus and Drosophila, BMP/Dpp ligands are inactivated by Chordin/Sog inhibitors at distal end and these complexes shuttle via diffusion back to the ligand source where inhibitors get cleaved, and ligands are released [61,132,133]. This shuttling mechanism, or as employed in engineering active diffusion [134], accounts naturally for scaling [135] while establishing a self-organized gradient [136].

### 8.1. Morphogen principles—scaling phenomenon

— Morphogen gradients can maintain their positional information content proportional with varying tissue sizes during development. This scaling phenomenon is usually transient.
— Tissue-wide clearance of morphogens poses a theoretical risk for scaling. Size regulation of morphogen source zone (exponentially for large tissues, linearly for short tissues) can maintain approximate scaling.
— Large tissues further exhibit an effective localization of sink to distal end from morphogen source. In ligand–receptor systems, this is accomplished in multiple ways such as negative feedback on receptor expression, and via extra molecules facilitating reduction of co-receptor activity, ligand recycling or active diffusion.

## 9. Do morphogen gradients provide multiple positional information?

In 1966 [137], Waddington had emphasized the need to separate ‘organization’ of cells from their ‘differentiation’; however, that distinction has proven to be quite challenging for many cases of developmental patterning [138]. Morphogen gradients [139] are indicated to control germ layer separation [136,140], fate specification [141,142], border determination [75,143] and growth [70,129,144] of embryonic tissues. Many times, either these events happen hand-in-hand or a single response is the outcome of multiple morphogen signals. Advent of quantitative techniques [145] and non-destructive imaging tools [146,147] together with spatio-temporally controlled perturbations of signalling (by optogenetics [148], drugs or inducible genetic lines) pave the way to investigate how morphogen gradients signal information for a particular event.

A widespread assumption is that morphogen gradients have multiple threshold targets as conceptualized with three colours of the ‘French flag problem’ (figure 4*b,c*) [36]. Theoretically, a steady-state morphogen gradient spanning approximately 100 cells with ≥10^3^ fold concentration span can provide positional information with ≤3% error (i.e. attain as many as 30 distinct threshold positions [40]). Even for a 10-fold concentration span, approximately 10 high-to-low threshold targets appear plausible. However, the gradient information doesn't appear to be extracted so prudently in nature. Usually, short-range (high-threshold) and long-range (low-threshold) targets are identified for morphogens. Dpp (BMP homologue) gradient in Drosophila dorsal ectoderm has three threshold targets: *race*, *tailup* and *pannier* in high-to-low order [149]. However, domains of *tailup* and *pannier* are organized combinatorial with opposing Brinker (Brk) gradient, which itself is repressed by Dpp signalling [149]. Likewise, short-range and long-range targets of wing disc Dpp gradient *spalt* and *optometric blind* [150] are both repressed by opposing Brk gradient [151]. In the same vein, Wingless (Wg, Wnt homologue) gradient of wing disc has three nested zones of targeted gene expression: *senseless*, *eutralized*, *achaeta* in cells next to the source stripe, *distalless (Dll)* in a wider domain, and *vestigial (vg)* along the broad zone of wing pouch [52]. However, positive regulation of longest range target *vg* by Wg signalling both prerequisites Vg presence [144] and requires combined inputs from other pathways [152]. The case is similar for early Drosophila A-P patterning where the Bicoid gradient is known to anteriorly activate head gap genes *orthodenticle* (*otd*), *empty spiracles* (*ems*) and *buttonhead* (*btd*), and the pair rule gene *sloppy-paired1* (*slp1*) [153]. Further posteriorly, gap genes *giant* (*gt*), *hunchback* (*hb*), *Krüppel* (*Kr*) and more posterior *knirps* (*kni*) are also known Bicoid targets [154]. Although the gradient itself has enough positional information down to single-cell resolution along the A-P axis [155], both nested domains of anterior targets [153] and positioning of other gap genes are resulting from mutual repressive exclusion of those transcription factor domains [156]. Furthermore, flies with flat and low Bicoid expression still display nested patterning of head gap genes [157] whereas removal of their repressors in presence of Bicoid gradient shifts the posterior margins of head gap genes to the same location [153]. Interestingly, patterning of such non-canalized transgenic embryos become sensitive to variations of embryonic geometry [158]. These observations highlight the importance of downstream regulatory network for patterning, which will be discussed further below.

Moving to vertebrates, transcriptional targets of mesendoderm inducer Nodal gradient emanating from yolk margin in zebrafish and expanding towards animal pole over time are also categorized as short and long-range [159]. Although that fits into multiple threshold targets perspective, long-range targets of Nodal can either actually be targets of Fgf signalling [160,161] which Nodal switches on [162] or merely manifest faster reaction kinetics as broader expression domains [163]. Similar concerns are valid for BMP signalling during vertebrate D-V patterning. Two recent works aimed to identify direct transcriptional targets of BMP signalling using two neatly designed alternative approaches and obtained sets of genes with various expression domains in zebrafish blastula: Mullins Lab [164] identified 29 ventrally expressed direct targets for BMP gradient which lack expression in mutants and were not known as common targets for other signalling pathways. Alternatively, Müller Lab [165] identified 16 high-confidence BMP targets (13 targets overlapped between two studies). Rogers *et al.* [165] succeeded to measure both transcriptional timing and spatial expression profiles for 9 of these targets, 7 of which also exist in Greenfeld *et al*.'s [164] identified target group. Subsets of genes switching on earlier in development also mostly displayed broader expression domains [165]. Interestingly, although expansion of pSmad5 gradient in Chordin mutants did not annihilate nested expression profile of targets [164], combined inhibition of two other pathways (Nodal and Fgf), in addition to expanding the pSmad1/5/9 gradient, vastly eliminated spatial differences between BMP target genes [165].

Examples can further be increased with other morphogens. This perspective suffices to suggest that classical multiple thresholds view of positional information is not demanded for morphogens. Here we focused on gene expression profiles. It's unusual to expect all known targets of any signalling molecule to display same exact spatial profile for a given input gradient. Rather, many factors such as time delays, halflives, time scales of downstream signalling network, feedback loops, cross-regulation by other pathways, and importantly ‘how the gradient is interpreted’ would result various spatial profiles. Therefore, these variations in spatial profiles do not necessitate morphogen gradients to positionally inform a process at multiple threshold points. Morphogens can still provide positional information at a single point [126].

### 9.1. Morphogen principles—positional information

— Morphogen gradients can theoretically exhibit enough information content to instruct numerous positions. Also, multiple target genes of a morphogen signalling pathway can exhibit nested expression patterns in a tissue. However, morphogen gradients do not appear to provide positional information to cells at numerous locations.
— Cells responsive to morphogens do not appear to be informed by a constant threshold of gradients.

## 10. What feature of a morphogen gradient provides positional information?

Although Wolpert's ‘French Flag Model’ proposed positional information to be encoded at constant concentration thresholds of signalling gradients, there is little evidence for a concentration threshold detection mechanism in any pathway studied so far. For example, concentration thresholds of Bicoid gradient were thought to be instructive for pattern formation. Therefore, much emphasis was given to its precision and scaling in fly embryos [139]. However, *bcd* heterozygote embryos survives. Embryos with different *bcd* copy numbers, displaying gradients differing up to fivefold in their maximal concentrations, also survive to fertile adults [41,158,166,167]. Embryos with flattened Bcd levels are able to express anterior target genes in almost correct positions and order. In these transgenic embryos, Bcd level is much lower than that in wild-type embryos suggesting constant Bcd concentrations are not needed for patterning [168]. Current evidence do not support the constant concentration model; temporally controlled perturbation studies instead suggest that Bcd levels might be integrated over time for anterior patterning [68,169]. More work is needed to determine how Bcd encodes positional information.

Shh signalling plays critical roles during neural tube patterning. In Shh mutant embryos, neural tube patterning is disrupted and ventral cell types are lost. However, these defects can be mostly recovered in double-mutant embryos lacking Shh and Gli3 [170,171]. Thus, similar to Bcd, absolute levels of Shh/Gli activity do not appear to be sufficient to determine gene expression patterns [172].

**Figure 5.**
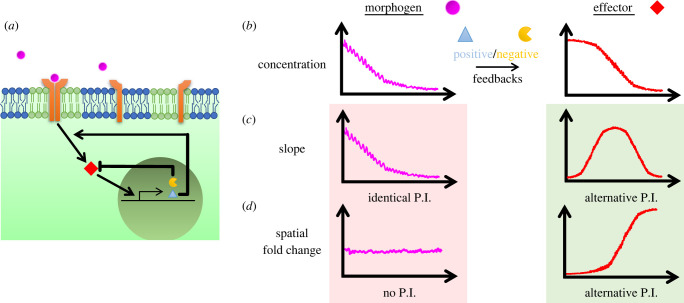
From morphogens to signalling. (*a*) Levels of receptor (orange) bound morphogens (magenta) are robustly relayed to downstream effector molecules (red). signalling pathways trigger transcription of negative (yellow) and positive (light blue triangle) feedback targets. (*b*) Exponential-like morphogen gradients (left) create sigmoidal-like effector gradients (right) under the influence of feedback molecules. (*c*) Slope of the morphogen gradient is proportional to the levels itself (i.e. identical positional information (P.I.), left). Slope of effector gradient peaks in midway and decreases back moving over space (right). (*d*) Spatial fold change (SFC) of morphogen gradient is approximately straight (i.e. no P.I., left). SFC of effector gradient increases moving away from the source (right).

Signalling pathways feature bottleneck effectors which can relay stimulus (morphogen) levels reliably and almost linearly for a broad range of concentrations [39] (figure 5*a*). That however does not require morphogen gradients to be interpreted with constant threshold values of effectors for a patterning task they inform. All of the broadly used pathways involve positive and negative feedback loops which contribute to robustness of signal relay [173,174] (figure 5*a*). We want to focus here on a different outcome of feedback loops: Although morphogen gradients are exponential due to diffusion-based mechanisms, effector gradients are non-exponential (e.g. sigmoidal-like with local maxima and an inflection point, figure 5*b*) due to feedbacks. Additionally, as previously discussed, many morphogens provide positional information on the go [169] and feedback loops can create a temporal ‘memory’.

At this point, it is possible to consider alternative mechanisms by which morphogen gradients might encode positional information: 1- persistence (i.e. temporal integration), 2- rate of change (i.e. temporal derivative or its fold change, adaptive response), 3- slope of the gradient and lastly 4- ratio among neighbouring cells, i.e. spatial fold change (SFC). This SFC detection among neighbouring cells ($s_{2}/s_{1}=1+\Delta s/s$) is mathematically similar to relative signal difference among cells ((*s*_2_ − *s*_1_)/*s*_1_), e.g. used in [175]. Assuming uniform cell density within the tissue of consideration along the morphogen gradient axis, SFC can also be formulated as the ratio of gradient slope to the signal itself, i.e. relative slope measurement (*m_s_*/*s*, where the slope $m_{s}=\Delta s/\Delta x$, e.g. used in [129], see also references in box 2). However, note that, biologically, the former detection mechanisms are cell-non-autonomous whereas the latter can be cell-autonomous. We should also emphasize here some information encoding alternatives listed above are only meaningful for effector gradients under the influence of feedback loops. For instance, because the slope of an exponential morphogen gradient will not be much different from the gradient itself, it cannot be an alternative mechanism for providing positional information (figure 5*c*). Likewise, SFC of an exponential morphogen gradient will be constant along space and thus will fail to provide any positional information (figure 5*d*). Those readouts bear alternative positional information contents only by the non-exponential shapes of effector gradients.

Box 2. **Random walks and fold change detection.**

The concept of fold change detection (FCD) emerged in molecular biology while studying bacterial chemotaxis and is studied exhaustively for temporal fold change [176]. Bacteria find their way to food sources by moving along the chemoattractant gradient. In the absence of a gradient, their linear motion interrupted with tumbling and random direction switches can be described as uniform spread of population over time with Einstein's diffusion law [63] (i.e. random walk [177]). Having random directions of motion with an average mean free path at first thought like a zero-sum game for displacement. Thinking about probabilities would elucidate the correct intuition here: the chances of a drunk person leaving home and getting back after a while is significantly lower than arriving at another place. The more time passes, the higher are the chances to be far away. However, as easily guessed, this motion doesn't land one towards a specific location. How do bacteria accomplish this with described walks? When we connect a metal wire to battery, applied potential difference decreases the chances of a free electron in wire to bump into another one if it were randomly moving towards lower potential. Although the direction needs not to be biased towards the negative terminal, longer mean free path this way with respect to the opposite results the current. Max Delbruck in 1970s thought what chemoattractant gradient controls is the same: how frequently bacteria tumbles [178]. Along the gradient, bacteria tumble more frequently moving downhill (i.e. shorter stretches of swimming, and less frequently uphill [112]).

Aggregation of slime mold Dictyostelium myxamoeba cells also happens chemotactically by sensing a diffusive substance gradient [179]. Relative concentration changes in space (between back and front ends of the cell) is proposed as gradient readout—estimated as approximately 5%—for slime mould [179]. In 1971, Keller and Segel, inspired by the prevalence of Weber-Fechner Law [180] in biological realm, proposed this relative slope (*Δ*s/s, mathematically identical to SFC) detection as the simplest mathematical solution explaining travelling bands phenomenon of bacterial chemotaxis [181]. Relative slope detection comes with a unique prediction for gradient responses: Bacteria should not have responded to an exponentially decaying gradient as discussed above (positional information is lost). This unique prediction of relative slope model was indeed verified experimentally [182]. However, for bacteria more than hundred times smaller than myxamoeba, relative slope detection between its front and back end relies on a minute approximately 10^–4^ change. Beautifully designed experiments of Macnab and Koshland replacing chemotactic gradient for bacteria in space with temporal gradient (rate of change) provided the answer: Bacteria were instead sensing temporal fold-change over their fast swim stretches resulting sensibly high signal changes from one tumbling to the next [183].

For developmental patterning, there is currently significant evidence for temporal fold-change (TFC) detection being widely at work. Notably, only two network motifs provide adaptive response in time [184] and an incoherent feedforward loop with one slow and one fast responding arms of network allow TFC detection [185]. Some examples are Wnt [186], EGF [187], Nodal [142] pathways and wing disc growth control by Dpp signalling [129]. Last case has a peculiarity: In 2005, Rogulja *et al*. had proposed proliferation of wing disc cells actually responds to the slope of the Dpp gradient which is measured not within a cell but as signal difference between neighbours [70]. Mosaic experiments showed cell non-autonomous effect on the growth in which cells responded signal changes of their neighbours [70]. However, stage-dependent amplitude increase disfavours slope detection for growth as the slope also increases with signal amplitude [129]. On the other hand, a uniform tissue growth can be maintained by using a fold-change detection because the fold-change of an exponential gradient is constant along a tissue. Wartlick and colleagues realized TFC of increasing Dpp signal (approx. 48% change over time) correlates with cell divisions driving growth [129]. Although the relative slope (i.e. SFC) alternative was also discussed in the supplementary material of Wartlick *et al*. [129], it was criticized as falling short to generate gradient scaling beyond a stage. However, beyond that stage, the Dpp gradient does not scale with tissue size [69]; Dpp signalling is not required for growth to continue [98]. Thus the SFC mechanism might be superior to TFC mechanism in explaining both the cell non-autonomous effect on the growth [70] and non-scaling gradient at later stages [69]. Unlike the slope itself, SFC detection would be insensitive to the amplitude changes and can still account for cell non-autonomous effects if cells are comparing not the signal difference but the ratio with their neighbours, i.e. the SFC mechanism (s_2_/s_1_ = 1 + Δs/s). As wing disc growth relies on difference of signals among neighbours [71], case for SFC detection should be re-evaluated.

**Figure 6.**
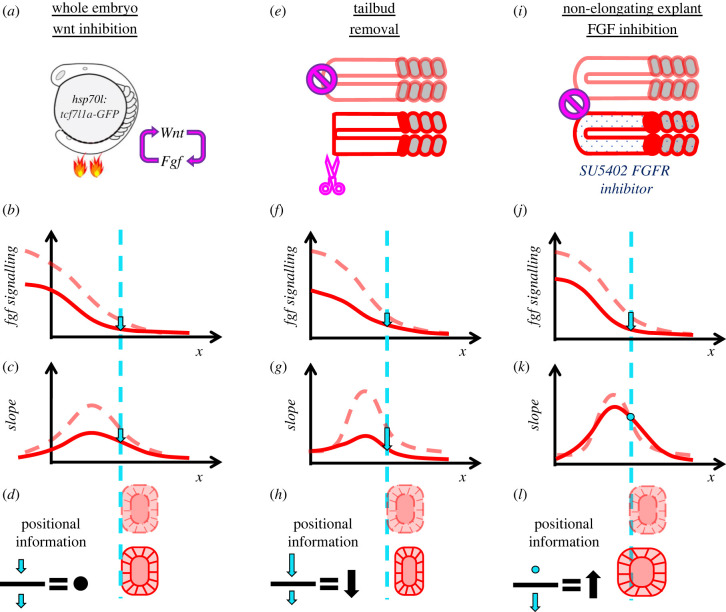
SFC detection mechanism for somite size determination. (*a*) Temporally controlled inhibition of Wnt signalling in whole embryos by heat shock promoted expression of dominant negative TCF transcription factors. Wnt and Fgf signalling are cross activating each other. (*b–d*) Both the levels (*b*) and the slope (*c*) of Fgf signalling drop proportionally one hour after Wnt inhibition (solid red) in comparison to unperturbed embryos (dashed light red). Proportional decrease of slope and levels (cyan arrows) leave SFC (slope over signal ratio, black) unchanged resulting in determination of normal somite sizes (*d*). Wnt inhibition, with another hour delay, eventually results in bigger somites as Fgf inhibition does (not shown). (*e*) Tail bud removal (red) eliminates cells actively transcribing Fgf ligands besides arresting axis elongation (light red control). (*f-g*) After tailbud removal, the slope (*g*) of Fgf signalling reduces more drastically than signalling levels do (*f*). (*h*) SFC at control location is lower after perturbation (black arrow), resulting in smaller somites. (*i*) Receptor inhibition of Fgf signalling with SU5402 drug in non-elongating explants. (*j*) Fgf signalling drops under the influence of drug treatment (solid red) below the case for non-elongating explant controls (dashed light red). (*k*) Slope of Fgf signalling remains still under opposing effects of elongation arrest and drug inhibition. (*l*) SFC at control location is higher after perturbation (black arrow), resulting in bigger somites.

Discriminating between alternative signal detection mechanisms is particularly challenging when multiple pathways combinatorially act (i.e. the wisdom of the crowd [140,188]). For instance, during somitogenesis, RA, Fgf and Wnt signalling pathways all establish gradients along the PSM. Both Fgf [75,76] and Wnt [189] form gradients from posterior tailbud towards anterior somites and perturbation of each pathway changes somite sizes. Furthermore, Fgf and Wnt signalling cross activate each other [78,189]. By contrast, RA signalling display a gradient in opposite direction. But, perturbation of RA signalling does not affect somite sizes in zebrafish [78]. Because Fgf and Wnt gradients are entangled and both regresses over PSM cells due to axis elongation, it had been difficult to discern between discussed alternative readouts for somite size determination by focusing on single-stage wild-type embryos. In our 2018 study, we devised elongation-arrested three-dimensional PSM explants which let us to test alternative readout detections. We found that only SFC detection mechanism could explain both explant and whole embryo data from various stages and under different perturbation conditions (figure 6). Moreover, using time controlled and localized perturbations, we showed somite sizes are determined cell non-autonomously by the SFC readout. Resolving detection mechanism also let us to untangle the effect of Wnt signalling on somite sizes. In the case of Wnt inhibition, status was opposite to elongation arrest experiments. Wnt inhibition was able to change Fgf signalling levels fast enough (figure 6*a,b*), however its effect on somite sizes came later only once SFC of Fgf signalling changed [78]. Wnt inhibition initially resulted proportional drops of both levels (figure 6*b*) and slope (figure 6*c*) of Fgf signalling, leaving SFC unchanged for a while (figure 6*d*). Likewise, we observed two experiments, both reducing Fgf signalling, resulting opposite outcome for somite sizes: while tail bud (Fgf source) removal in explants resulted smaller somites (figure 6*e–h*), SU5402 (Fgf receptor inhibition) treatment in explants resulted bigger ones (figure 6*i–l*). These contrasting results are also explainable with the SFC model: Receptor inhibition decreases the signal (figure 6*j*) whereas opposing effects of signal inhibition and elongation arrest leave slope unchanged (figure 6*k*), causing SFC to increase and bigger somites to form (figure 6*l*). However, removal of the tailbud decreases the slope more significantly (figure 6*g*) than the levels of gradient (figure 6*f*). As a result, SFC decreases and shorter somites form (figure 6*h*). SFC detection might be more widely used during development. For instance, blastula cells correct for Wnt gradient noise by comparing values between neighbouring cells and eliminating outfit ones [175]. Mathematical formulation of this cell competition response depends on SFC (*Δ*s/s) values. Recently, SFC detection of Activin signalling, named the ‘neighbourhood watch model’, is also proposed for positioning the site of primitive streak formation in chicks [190].

### 10.1. Morphogen principles—relay of signalling

— Downstream effector molecules of diffusive morphogen signals can maintain non-exponential gradients due to signalling pathway feedback modules.
— Morphogen gradients can relay their positional information content at a precise location through effector molecules via numerous alternative methods, including persistence, rate of change, gradient slope and spatial fold-change (neighbour comparison) of the signal.
— Fold-change detection in either time or space appears a widespread, fast-acting and reliable method to extract positional information from morphogen gradients.

Although fold-change detection mechanisms are advantageous to measure changes while eliminating basal signalling, other slowly evolving patterns might need measurement of persistence and eliminate signal fluctuations. This can be accomplished by a pathway through negative feedbacks integrating signal over time as proposed for Shh signalling [191] or a persistent pool of effector staying in nucleus as proposed for Activin signalling in *Xenopus*. Elucidation of detection mechanisms are crucial to understand how morphogen gradients relay their vital information [192]. As a handful of pathways are recursively used during metazoan development, it is apprehensible to expect same pathways utilize various detection mechanisms for different tasks. Focusing on informative changes for a specific biological question can lead the way in uncovering patterning principles with morphogens.

## Data Availability

This article has no additional data.

## Glossary

Blastula: An early developmental stage of metazoan embryo before gastrulation which can be described as a ball of cells.
Gastrulation: Collective motion of blastula cells forming embryonic body axes and organized structure of three germ layers.
Animal vegetative axis: Fertilized egg cell starts mitotic divisions and develop into an embryo supported by a vegetative yolk tissue. Animal- vegetative axis is defined perpendicular to the yolk margin cells as blastula develops.
Embryonic body axes: A bilaterian embryo following gastrulation starts exhibiting mirror symmetry of development specifying a medio-lateral axis. Perpendicularly mouth and anus locations designate a head-to-tail anteroposterior axis. In the third orthogonal direction the dorsoventral axis is established as cells gastrulate medially and get displaced away from the vegetative yolk.
Polarity: Three-dimensional development of embryo from a single fertilized cell involves localized specification of cells or embryonic materials along the embryonic body axes. This asymmetric localization establishes polarity.
Positional information: Molecular information provided to embryonic cells indicating their location with respect to embryonic body axes and certain tissue landmarks (i.e. reference points).
Totipotent: An unspecified embryonic stem cell that is able to form any tissue within the embryo.
Limb bud: An early embryonic structure in vertebrate embryos emerging with local molecular interactions between ectoderm and underlying mesoderm layers, giving rise to lateral limbs with distal growth.
Blastopore lip: With gastrulation the blastula folds in and forms three germ layers. The blastopore lip is the margin of the blastula fold depicting the dorsal limit of the embryo.
Somite: Blocks of embryonic mesoderm tissue in vertebrates which segment bilaterally from the paraxial mesoderm in a sequential manner. Later in development, somites differentiate into muscles, vertebrae and skin. Intersomitic boundaries along the head-to-tail axis provide positional cues for development of the peripheral nervous system and angiogenesis.
